# Caspase-11/GSDMD contributes to the progression of hyperuricemic nephropathy by promoting NETs formation

**DOI:** 10.1007/s00018-024-05136-z

**Published:** 2024-03-04

**Authors:** Fan Wu, Caiming Chen, Guo Lin, Chengkun Wu, Jingzhi Xie, Kongwen Lin, Xingchen Dai, Zhengyue Chen, Keng Ye, Ying Yuan, Zhimin Chen, Huabin Ma, Zishan Lin, Yanfang Xu

**Affiliations:** 1 Department of Nephrology, Blood Purification Research Center, The First Affiliated Hospital, Fujian Medical University, Fuzhou, 350005 China; 2 Research Center for Metabolic Chronic Kidney Disease, The First Affiliated Hospital, Fujian Medical University, Fuzhou, 350005 China; 3 Department of Nephrology, National Regional Medical Center, Binhai Campus of the First Affiliated Hospital, Fujian Medical University, Fuzhou, 350212 China; 4 Central Laboratory, The First Affiliated Hospital, Fujian Medical University, Fuzhou, 350005 China; 5 Department of Intensive Care Unit, The First Affiliated Hospital, Fujian Medical University, Fuzhou, 350005 China; 6 School of Medicine, Nankai University, Tianjin, 300071 China

**Keywords:** Gasdermin D, Caspase-11, Neutrophil extracellular traps, Hyperuricemic nephropathy

## Abstract

Hyperuricemia is an independent risk factor for chronic kidney disease (CKD) and promotes renal fibrosis, but the underlying mechanism remains largely unknown. Unresolved inflammation is strongly associated with renal fibrosis and is a well-known significant contributor to the progression of CKD, including hyperuricemia nephropathy. In the current study, we elucidated the impact of Caspase-11/Gasdermin D (GSDMD)-dependent neutrophil extracellular traps (NETs) on progressive hyperuricemic nephropathy. We found that the Caspase-11/GSDMD signaling were markedly activated in the kidneys of hyperuricemic nephropathy. Deletion of *Gsdmd* or *Caspase-11* protects against the progression of hyperuricemic nephropathy by reducing kidney inflammation, proinflammatory and profibrogenic factors expression, NETs generation, α-smooth muscle actin expression, and fibrosis. Furthermore, specific deletion of *Gsdmd* or *Caspase-11* in hematopoietic cells showed a protective effect on renal fibrosis in hyperuricemic nephropathy. Additionally, in vitro studies unveiled the capability of uric acid in inducing Caspase-11/GSDMD-dependent NETs formation, consequently enhancing α-smooth muscle actin production in macrophages. In summary, this study demonstrated the contributory role of Caspase-11/GSDMD in the progression of hyperuricemic nephropathy by promoting NETs formation, which may shed new light on the therapeutic approach to treating and reversing hyperuricemic nephropathy.

**Supplementary Information:**

The online version contains supplementary material available at 10.1007/s00018-024-05136-z.

## Introduction

Chronic kidney disease (CKD) represents a leading global public health concern associated with high morbidity, mortality, and healthcare costs [1]. Hyperuricemia is thought to be tightly associated with the development and progression of CKD and is proven to be an independent risk factor for end-stage renal disease [2, 3]. Hyperuricemic nephropathy is characterized by non-specific vascular sclerosis, tubular atrophy, interstitial fibrosis, and foci of interstitial inflammation [4]. Traditionally, hyperuricemia has been thought to drive kidney injury by deposition of crystalluria, inciting inflammation [5]. Recent research has supplied evidence that hyperuricemia could also induce kidney injury through crystalluria-independent mechanisms involving endothelial dysfunction, oxidative stress, renin–angiotensin system activation, tubular epithelial-to-mesenchymal transition, and vascular smooth muscle cell proliferation [6–9]. In addition, uric acid can behave as a danger-associated molecular pattern stimulating the NLRP3/ASC/Caspase-1/IL-1β axis, thereby contributing to the progression of diabetic kidney disease [10]. Nonetheless, the precise mechanism by which hyperuricemia drives the progression of CKD remains elusive.

Irrespective of the underlying etiology, CKD is histologically characterized by tubulointerstitial fibrosis, marked by excessive accumulation and deposition of extracellular matrix proteins such as fibronectin and collagen I (Col I) [11]. In response to diverse insults, abundant inflammatory cells migrate and infiltrate into the tubulointerstitium, releasing proinflammatory and profibrotic factors, ultimately leading to renal fibrosis [11, 12]. Despite extensive investigations, the cellular and molecular mechanisms facilitating renal fibrosis remain incompletely understood.

Neutrophils have long been known as front-line soldiers of the non-specific immune system armed with limited proinflammatory duties [13]. Yet recently, another mechanism of neutrophils trapping and killing pathogens was reported and suggested a new term, namely, neutrophil extracellular traps (NETs) [14]. NETs, characterized by a network chromatin structure in the extracellular space and adorned with cytosolic and granule proteins, serve both infectious and non-infectious functions, including tissue injuries [14, 15]. Excessive release of NETs is strongly linked to various sorts of acute kidney injury, especially in the early injury phase [16]. However, the contribution of NETs to the subsequent development of renal fibrosis remains largely unexplored.

Emerging evidence underscores the pivotal role of Gasdermin D (GSDMD), the pyroptosis execution protein, in facilitating NETs generation during bacterial infection, sickle cell disease, and other pathological conditions [17–20]. While our prior study identified GSDMD-dependent NETs as an essential role in promoting obstructive nephropathy, the potential involvement of GSDMD and NETs in the development of hyperuricemic nephropathy remains unclear [20]. A recent study demonstrated that the expressions of GSDMD and Caspase-11 were markedly upregulated in the kidney of hyperuricemic nephropathy [21]. In the current study, we scrutinized the impact of Caspase-11/GSDMD and NETs on the progression of hyperuricemic nephropathy and delineated the associated mechanistic pathways.

## Materials and methods

### Mice

All the experiments were conducted according to the Chinese Guidelines on the Care and Use of Laboratory Animals approved by the Institutional Animal Care and Use Committee at Fujian Medical University (FJMU IACUC 2021-0298).

*Gsdmd*^*−/−*^ mice and *Gsdmd*^+*/*+^ mice were kindly provided by Prof. Jiahuai Han from Xiamen University. *Gsdmd*^*fl/fl*^ mice and *Caspase-11*^*fl/fl*^ mice were purchased from Cyagen Biosciences. *Vav-Cre* mice were purchased from Jackson Lab. The *Vav-Cre* mice was hybridized with *Gsdmd*^*fl/fl*^ mice and *Caspase-11*^*fl/fl*^ mice to generate *Vav-Cre Gsdmd*^*fl/fl*^ mice and *Vav-Cre Caspase-11*^*fl/fl*^ mice, respectively. Genotypes were identified by tail-snip PCR amplification (Supplementary Fig.1).

### Mice model of hyperuricemic nephropathy and sample collection

8–10-week-old male mice were fed with a diet containing 0.2% adenine (D190625, Dyets, China) [22]. Kidneys were harvested at the designated time points and cryopreserved with liquid nitrogen promptly. The blood was collected in coagulant tubesand centrifuged at 2500*g* (4 °C) for 15 min until serum was isolated. All samples were stored at −80 °C until further analyses.

### Histologic analysis

Renal tissue sections were deparaffinized and rehydrated. Masson’s trichrome or immunofluorescence staining was performed for assessment of kidney fibrosis. Thin sections were incubated with the different primary antibodies, including anti-collagen I (1:200; ab34710, Abcam), anti-α-SMA (1:200; A5228, Sigma-Aldrich), anti-Lrp2/Megalin (ab184676, Abcam), anti-Histone H3 (1:200; AF0863, Affinity Bioscience, China), anti-MPO (1:200; ab208670, Abcam), anti-F4/80 (1:200; ab6640, Abcam), anti-Ly6G (1:200; ab25377, Abcam), and anti-GSDMD (1:200; ab239377, Abcam) for 1–2 h, and then stained with Alexa Fluor® 594 or Alexa Fluor® 488 labeled secondary antibodies (Abcam). DAPI (D3571, Invitrogen) was used to indicate DNA. The Image J software was utilized for quantification of T Ly6G^+^, F4/80^+^, NETs^+^, and F4/80^+^α-SMA^+^ cells. Imaging was performed using the FPMRC-EasyScan Histology Slide Scanner and image morphology was analyzed using ImagePro Plus image analysis software.

### Cell culture

The neutrophils and macrophages were aseptically isolated from the bone marrow of mice as previously described [20]. In brief, the mice were euthanized and the bone marrow cells were isolated from the femur and tibia. Equal volumes of 52%, 62%, and 72% Percoll (17-0891-02, GE Healthcare, Sweden) were used for gradient centrifugation. The third layer (neutrophils) was collected and seeded in a 24-well tissue plate for treatment. To stimulate NETs in vitro, we treated neutrophils with PMA (20 nM) and then cultured with or without uric acid (5 mM) for 3 h. The bone marrow cells were cultured in RPMI 1640 (31800-089, Gibco™) medium supplemented with 30% L929. After a period of 6–7 days, bone marrow-derived macrophages were divided and collected.

### Immunocytochemistry

Cells for immunofluorescence were fixed with 2% paraformaldehyde and permeabilized with 0.15% Triton X-100. Then, cells were incubated with primary antibodies such as α-SMA, MPO, and Cit-H3 at room temperature for 3 h, followed by fluorescent secondary antibodies labeled with Alexa Fluor^®^ (488, 594, or 647) for 1 h. Nuclei were counterstained with DAPI. Expression of target proteins was observed by confocal microscopy (Leica DMI600, Leica SPE).

### Western blot analysis

Cells were lysed with 1.2 × SDS buffer. Each 50 mg of kidney tissue was homogenized in 300–500 μL of radioimmunoprecipitation assay (RIPA) lysis buffer with ethylenediaminetetraacetic acid and protease inhibitor cocktail (Thermo Fisher). The proteins were adjusted to a certain concentration by adding 5 × SDS buffer and RIPA lysis buffer. After electrophoresis and separation on polyacrylamide gels of 8–15%, the lysates were transferred to polyvinylidene fluoride membranes (PVDF, EMD Millipore). After blocking, the membrane was incubated with the primary antibodies (1:1000) overnight at 4 °C, including anti-GSDMD antibody (Rabbit polyclonal; ab219800, Abcam), anti-collagen I antibody (Rabbit polyclonal; ab260043, Abcam), anti-α-SMA antibody (Mouse monoclonal; A5228, Sigma-Aldrich), anti-Caspase-11 antibody (Rat monoclonal; 17D9, Vovus Bio), anti-mouse IL-1β antibody (Rabbit polyclonal; 5129-100, BioVision), anti-pSmad3 antibody (Rabbit polyclonal; ab52903, Abcam), and anti-GAPDH antibody (Mouse monoclonal; 60004, Proteintech). After washing, the membranes were incubated with horseradish peroxidase-labeled secondary antibodies (1:2000) for 1 h. The binding antibodies were visualized through enhanced chemiluminescence (ECL).

### Flow cytometry analysis

Briefly, kidneys were collected, minced, and incubated with DNase I (90083, ThermoFisher Scientific) and type IV collagenase (sigma, 11088858001) in RPMI 1640 at room temperature in a shaker for 30 min. The digestion was ended using RPMI and FBS (085-150, WISENT). The tissue suspension was milled into a single-cell suspension through a mesh with a 40 μm pore size. After blocking, the fresh suspensions were incubated with anti-mouse CD45 antibody (30-F11, 47-0451-82, APC-eFluor™ 780, eBioscience™) to ensure the number of immune cells. Then, the suspensions were labeled with Ly-6G/Ly-6C antibody (RB6-8C5, 11-5931-82, FITC eBioscience™) and anti-mouse F4/80 antibody (BM8, 48-4801-82, PE, eBioscience™) to identify neutrophils and macrophages, respectively.

### Real-time quantitative polymerase chain reaction

Real-time quantitative polymerase chain reaction (RT-qPCR) was conducted according to the previous protocol [23]. Kidney immune cells were extracted according to the previous method. RNA from renal immune cells was extracted separately using Trizol (Invitrogen, USA) reagent by the manufacturer's instructions and then transcribed into cDNA using a reverse transcriptase kit (Vazyme, R333-01). RT-qPCR was conducted using SYBR qPCR Mix as a fluorescent dye on QuantStudio 5 (Thermo Fisher Scientific). The primers involved in the current study were obtained from Metabion (Martinsried, Germany) and were listed as follows, IL-1β (5′-TGGAGAGTGTGGATCCCAAG-3′ and 3′-GGTGCTGATGTACCAGTTGG-5′), TNF-α (5′-AACTAGTGGTGCCAGCCGAT-3′ and 3′-CTTCACAGAGCAATGACTCC-5′), TGF-β1 (5′-ACCGCAACAACGCCATCTATGAG-3′ and 3′-GGCACTGCTTCCCGAATGTCTG-5′), HMGB1 (5′-GCTGACAAGGCTCGTTATGAA-3′ and 3′-ATGGCGGGGTTTTAGTTTCC-5′), and β-actin (5′-GGCTGTATTCCCCTCCATCG-3′ and 3′-CCAGTTGGTAAC AATGCCATGT-5′).

### Enzyme-linked immunosorbent assay

Kidney tissue is milled on ice in a 2 mL Eppendorf tube containing RIPA buffer and protease inhibitors. After standing for half an hour to lyse, the supernatant was collected after centrifugation at 12,000 rpm at 4 °C for 20 min and then analyzed with an enzyme-linked immunosorbent assay (ELISA) kit. The secretion levels were assessed by ELISA kits including mouse IL-1β (MLB00C), TNFα (MTA00B), TGF-β1 (DB100C) from R&D Systems, and HMGB1 (ST51011) from TECAN.

### Statistical analysis

GraphPad Prism software (GraphPad Software, Inc.) was used for image rendering and data analyzing. All data were presented as the mean ± SD values. Unpaired Student’s *t* tests or two-way analysis of variance test with Bonferroni post-test were performed to compare the means of the two groups. A *P* value less than 0.05 was considered to be statistically significant.

## Results

### *Gsdmd* deficiency alleviated renal fibrosis in hyperuricemic nephropathy

**Fig. 1:**
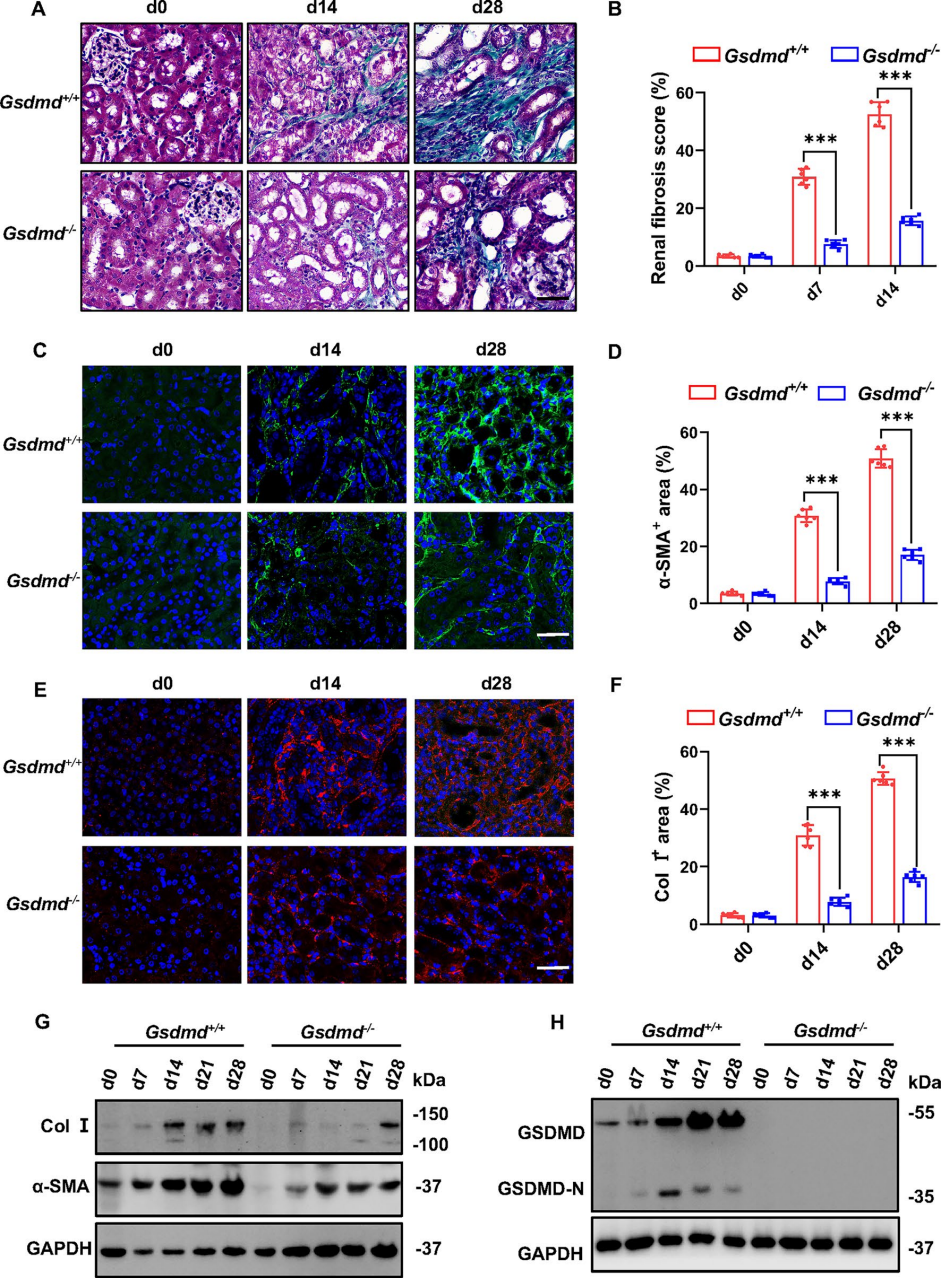
*Gsdmd* deficiency alleviated renal fibrogenesis in the hyperuricemic nephropathy model. **A** Representative microphotographs of Masson trichrome staining in mice kidney cortical tissue after daily adenine feeding for 0, 14, and 28 days. Scale bar = 100 μm. **B** Renal fibrosis scores assessed by Masson trichrome staining. *n* = 6. **C, E** Representative immunofluorescence images stained with α-SMA (**C**) and Col I (**E**) in mice kidney cortical tissue. Scale bar = 100 μm. **D, F** Quantitative analysis of α-SMA (**D**) and Col I (**F**) expression. *n* = 6. **G** Representative Western blot images, showing the expression of α-SMA and Col I expression in kidney tissue. Kidney tissue lysates were isolated on day 0, 7, 14, 21, and 28 after daily adenine feeding. *n* = 4. **H** Representative Western blot images, showing the expression of GSDMD and its cleaved form, GSDMD-N, in kidney tissue. *n* = 4. ***P < 0.001

In the context of CKD, the development of renal fibrosis represents a pervasive pathological phenomenon, irrespective of its underlying etiology. To explore the role of *Gsdmd* in the progression of renal fibrosis in hyperuricemic nephropathy, we compared the degree of renal fibrosis in *Gsdmd*^+*/*+^ mice and *Gsdmd*^*−/−*^ mice after daily feeding of adenine for 14 days and 28 days. Compared to *Gsdmd*^*−/−*^ mice, the kidneys of *Gsdmd*^+*/*+^ mice displayed more collagen deposition within the tubulointerstitium, as evidenced by Masson Trichrome staining (Fig. 1A, B). Consistently, expression of fibrotic markers, α-smooth muscle actin (α-SMA), and Col I was upregulated in the kidneys of *Gsdmd*^+*/*+^ mice, and *Gsdmd* deficiency reduced their expression (Fig. 1C–G). Upon subjecting mice to adenine feeding, the expression and cleaved levels of GSDMD in the kidney of *Gsdmd*^+/+^ mice were also elevated compared to those of *Gsdmd*^*−/−*^ mice (Fig. 1H). These findings collectively underscore the contributory role of GSDMD in the progression of renal fibrosis associated with hyperuricemic nephropathy.

### *Caspase-11* deficiency alleviated renal fibrosis in hyperuricemic nephropathy

**Fig. 2:**
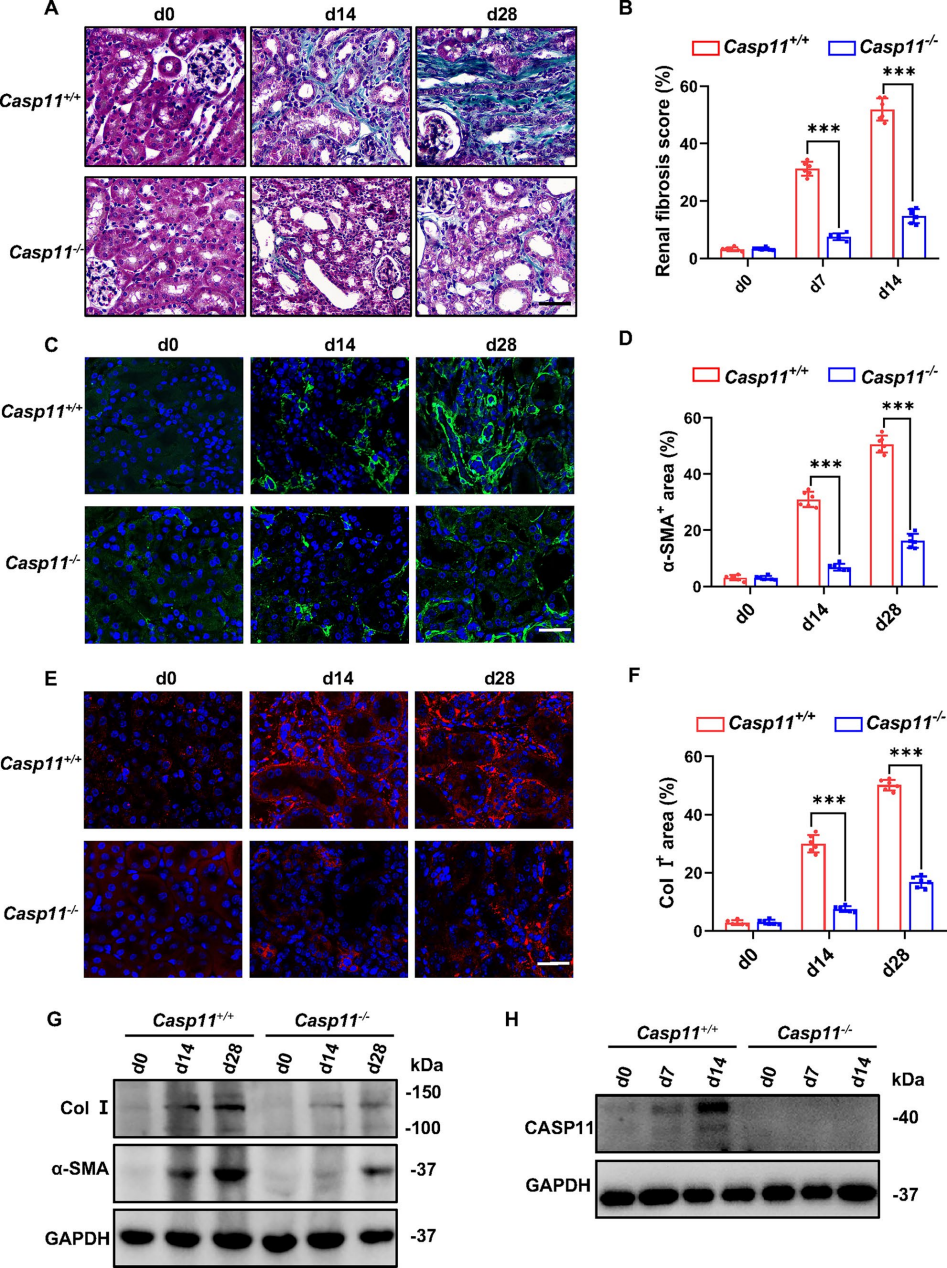
*Caspase-11* deficiency alleviated renal fibrogenesis in the hyperuricemic nephropathy model. **A** Representative microphotographs of Masson trichrome staining in mice kidney cortical tissue. Kidneys were harvested from wild-type mice (*Caspase-11*^+/+^) and *Caspase-11*-deficient mice (*Caspase-11*^*−/−*^) after daily adenine feeding for 0, 14, and 28 days. Scale bar = 100 μm. **B** Renal fibrosis scores assessed by Masson trichrome staining. *n* = 6. **C, E** Representative immunofluorescence images stained with α-SMA (**C**) and Col I (**E**) in mice kidney cortical tissue. Scale bar = 100 μm. **D, F** Quantitative analysis of α-SMA (**D**) and Col I (**F**) expression. *n* = 6. **G** Representative Western blot images, showing the expression of α-SMA and Col I in kidney tissue, which were isolated on days 0, 14, and 28 after daily adenine feeding. *n* = 4. **H** Representative Western blot images, showing the expression of Caspase-11 in kidney tissue. Kidney tissue lysates were isolated on day 0, 7, and 14 after daily adenine feeding. *n* = 4. ***P < 0.001

As is well established, caspases, including Caspase-1, 4, 5, and 11 could cleave GSDMD [24]. Our previous study demonstrated that both Caspase-11 and GSDMD were activated and contributed to renal fibrosis in obstructed kidneys [20]. We sought to extend our inquiry into the involvement of Caspase-11 in renal fibrosis in the murine model of hyperuricemic nephropathy. As expected, *Caspase-11* deficiency reduced the positive fibrosis area of Masson’s trichrome staining, as well as the expression levels of a-SMA and Col I (Fig. 2A–G). The knockout efficiency of *Caspase-11* was duly confirmed (Fig. 2H). These findings substantiate that the deletion of Caspase-11 serves to alleviate renal fibrosis in the context of hyperuricemic nephropathy.

### *Gsdmd* deficiency diminished inflammatory cell infiltration in hyperuricemic nephropathy

**Fig. 3:**
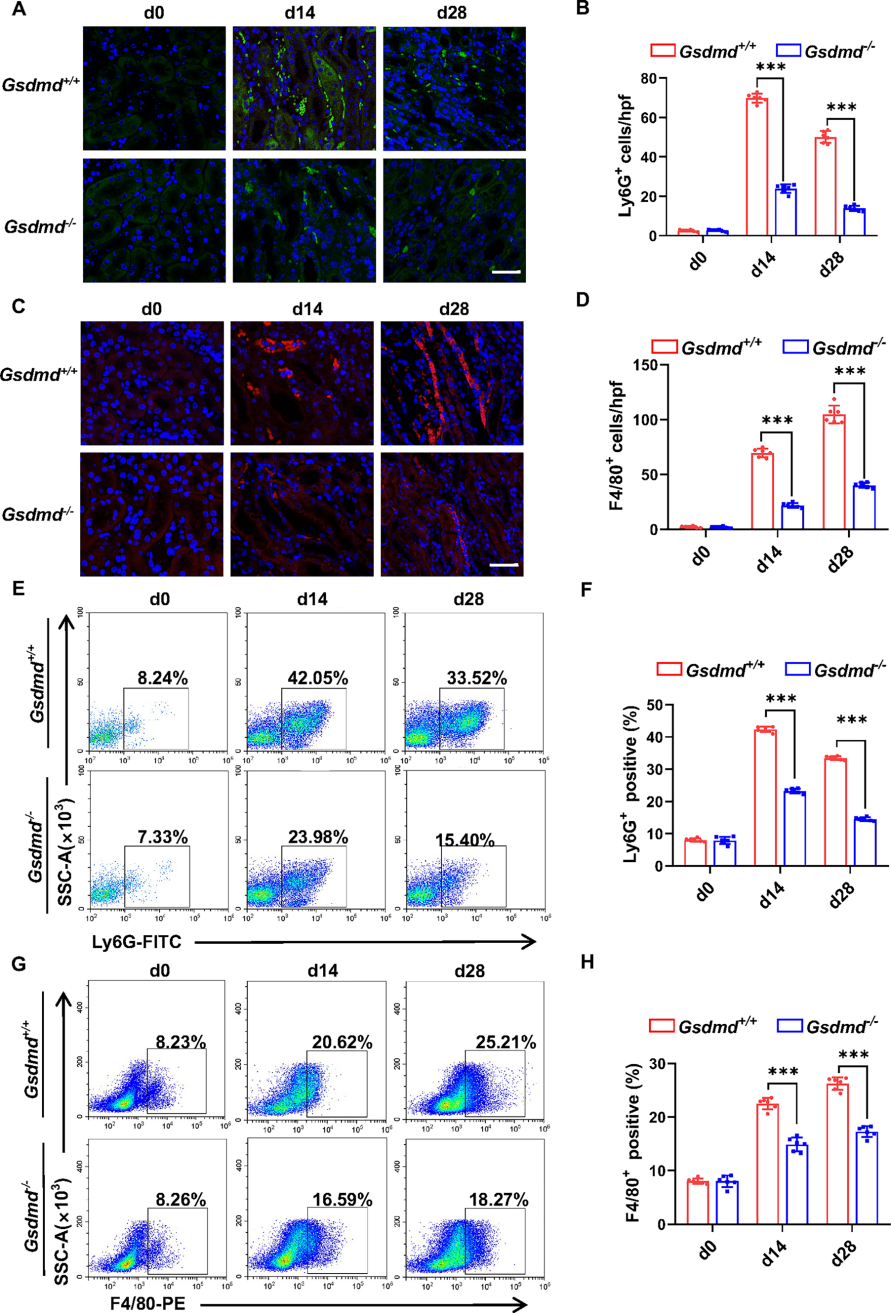
*Gsdmd* deficiency diminished inflammatory cell infiltration in the hyperuricemic nephropathy model. **A, C** Representative immunofluorescence images stained with Ly6G **A** and F4/80 **C** in mice kidney cortical tissue. Scale bar = 100 μm. **B, D** Positive staining cells of Ly6G (**B**) and positive staining cells of F4/80 (**D**) were calculated and graphed. *n* = 6. **E, G** Representative flow cytometry results of Ly6G (**E**) and F4/80 (**G**) in the kidney. **F, H** Quantitative analysis of the percentage of neutrophils (Ly6G^+^) and macrophages (F4/80^+^) infiltration. *n* = 6. ***P < 0.001

Inflammatory cell infiltration constitutes a notable pathological change observed across various forms of CKD, including hyperuricemic nephropathy [25]. Moreover, aligning with the prior study, our study noted an elevation in GSDMD within the peritubular compartment following adenine treatment, with no discernible increase in proximal tubular cells (Supplementary Fig. 2), suggesting GSDMD in inflammatory cells were involved in hyperuricemic nephropathy [26]. In an effort to unravel the effects of *Gsdmd* on renal inflammation, we conducted immunofluorescence staining and flow cytometry analyses. Immunofluorescence staining revealed a surge in the number of neutrophils and macrophages, identified by Ly6G^+^ cells (Fig. 3A, B) and F4/80^+^ cells (Fig. 3C, D), after daily feeding of adenine for 14 and 28 days. Notably, these increments were all markedly mitigated by the deletion of *Gsdmd*. Flow cytometry analyses further substantiated these findings, revealing an emergence of inflammatory cell recruitment in the kidneys of *Gsdmd*^+*/*+^ mice following daily adenine feeding, whereas this response was significantly attenuated in *Gsdmd*^*−/−*^ mice (Fig. 3E–H).

### *Caspase-11* deficiency diminished inflammatory cell infiltration in hyperuricemic nephropathy

**Fig. 4:**
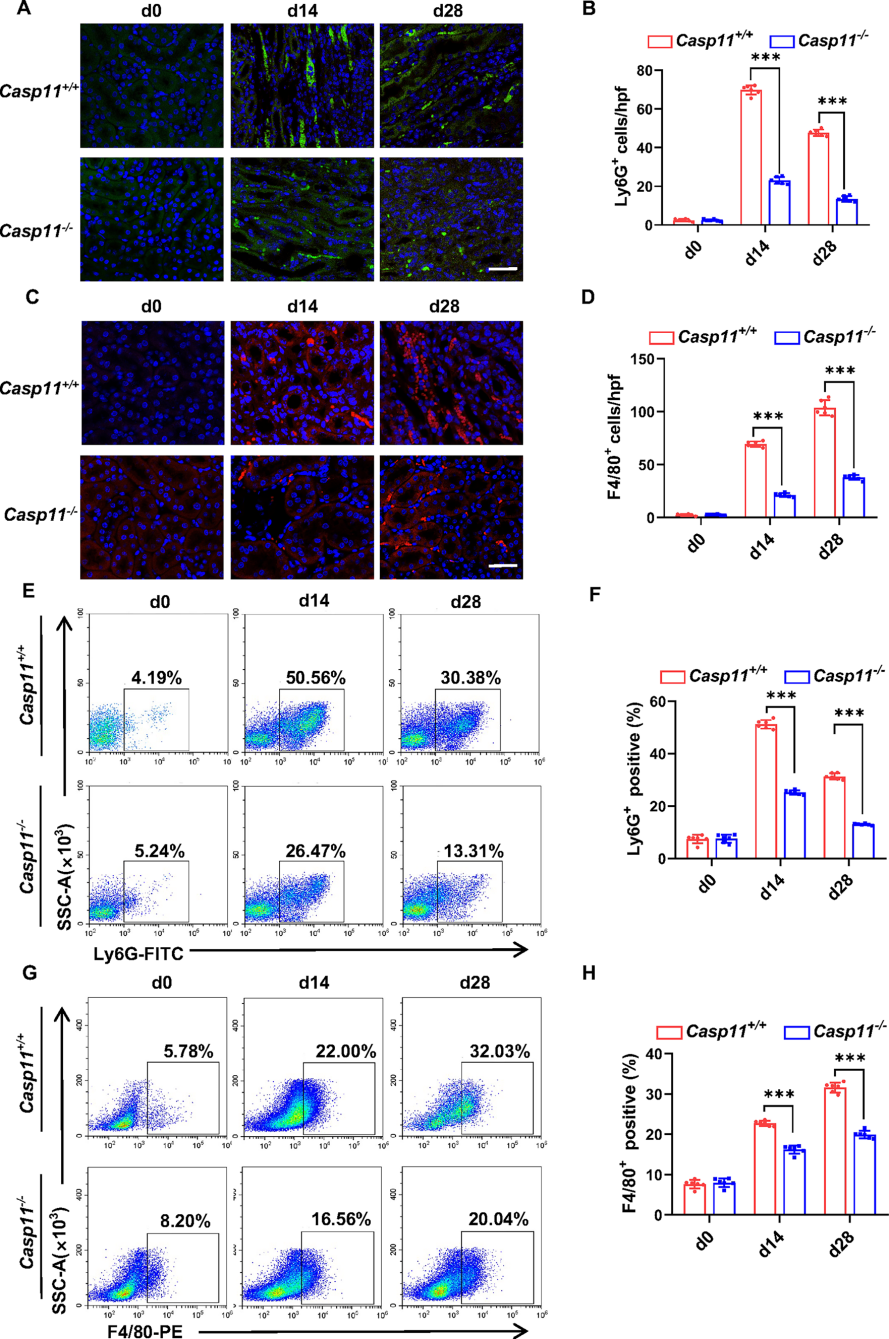
*Caspase-11* deficiency diminished inflammatory cell infiltration in the hyperuricemic nephropathy model. **A, C** Representative immunofluorescence images stained with Ly6G (**A**) and F4/80 (**C**) in mice kidney cortical tissue. Scale bar = 100 μm. **B, D** Positive staining cells of Ly6G (**B**) and positive staining cells of F4/80 (**D**) were calculated and graphed. *n* = 6. **E, G** Representative flow cytometry results of Ly6G (**E**) and F4/80 (**G**) in the kidneys. **F, H** Quantification of the percentage of neutrophils (Ly6G^+^) and macrophages (F4/80^+^) infiltration. *n* = 6. ***P < 0.001

Next, we evaluated the effect of *Caspase-11* on renal inflammation in the context of hyperuricemic nephropathy. Following 14 days and 28 days of daily adenine feeding, the number of Ly6G^+^ cells and F4/80^+^ cells in the injured kidney was significantly increased in *Caspase-11*^+*/*+^ mice, and depletion of *Caspase-11* significantly reduced their infiltration (Fig. 4A–H). Altogether, our data indicates that the activation of GSDMD/Caspase-11 is imperative for the infiltration of neutrophil and macrophage in kidney tissue in the setting of hyperuricemic nephropathy.

### Caspase-11/GSDMD played a vital role in the expression of proinflammatory and profibrogenic factors, and the production of α-SMA in macrophages

**Fig. 5:**
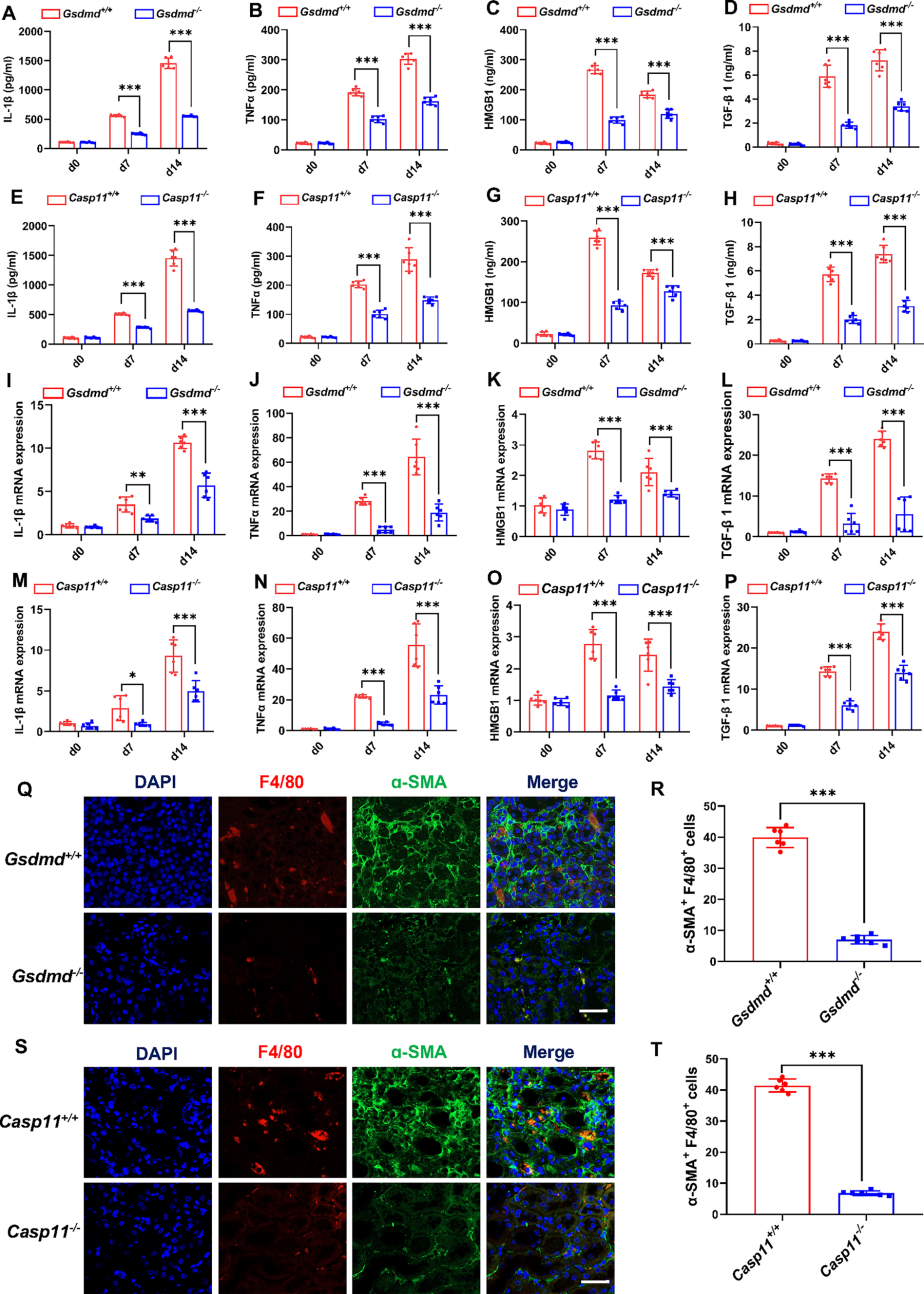
Deletion of *Caspase-11/Gsdmd* reduced the expression of proinflammatory and profibrogenic factors, and the production of α-SMA in macrophages in the hyperuricemic nephropathy model. **A**–**H** Expression level of IL-1β (**A, E**), TNFα (**B, F**), HMGB1 (**C, G**), and TNF-β1 (**D, H**) production in kidneys were evaluated by ELISA. Kidneys were harvested from *Gsdmd*^+*/*+^ mice, *Gsdmd*^*−/−*^ mice, *Capase-11*^+*/*+^ mice, and *Caspase-11*^*−/−*^ mice after daily adenine feeding for 0, 7, and 14 days.* n* = 6. **I**–**P** Real-time quantitative polymerase chain reaction analysis revealed the mRNA expression level of IL-1β (**I, M**), TNFα (**J, N**), HMGB1 (**K, O**), and TNF-β1 (**L, P**) in immune cells isolated from kidneys. *n* = 6. **Q, S** Representative images of immunofluorescence staining showing macrophage-to-myofibroblast transition cells that coexpress F4/80 and α-SMA in mice kidneys on day 28 after daily adenine feeding. Scale bar = 100 μm. **R, T** Quantification of cells coexpressing F4/80 and α-SMA by immunofluorescence. *n* = 6. *P < 0.05. **P < 0.01. ***P < 0.001

Given the pivotal role of generation and activation of proinflammatory and profibrogenic factors in the progression of renal fibrosis, we probed into the influence of Caspase-11/GSDMD on the generation of the selected factors, including interleukin-1beta (IL-1β), tumor necrosis factor-alpha (TNFα), transforming growth factor-beta1 (TGF-β1), and high-mobility group box-1 (HMGB1) in the kidney by ELISA. Our data suggested that expression of these factors was significantly upregulated in the kidney of hyperuricemic wild-type (WT) mice and significantly downregulated by depletion of *Gsdmd* or *Caspase-11* (Fig. 5A–H). We further detected the expression of these factors released by immune cells in the kidneys. Consistent with expectations, the RT-qPCR array analysis indicated a significant reduction in the expression of IL-1β, TNFα, TGF-β1, and HMGB1 in immune cells upon the depletion of *Gsdmd* or *Caspase-11* (Fig. 5I–P). These observations underscore the regulatory role of Caspase-11/GSDMD in modulating the expression of key inflammatory and fibrotic factors within the renal microenvironment in hyperuricemic nephropathy.

Myofibroblasts, recognized as the primary producers of kidney collagen, have been identified as pivotal contributors to renal fibrosis. Recent research has provided evidence supporting the notion that macrophages can undergo direct transdifferentiation into myofibroblasts through a process termed macrophage-to-myofibroblast transition (MMT), thereby actively participating in the progression of renal fibrosis [27]. To identify MMT cells in hyperuricemic nephropathy, we sought to detect cells expressing myofibroblast (α-SMA) and macrophage (F4/80^+^) markers. Consistent with the previous study, a substantial number of F4/80^+^α-SMA^+^ cells featuring active fibrosis, were observed in the renal interstitium of hyperuricemic WT mice at day 28 (Fig. 5Q–T). In contrast, the occurrence of MMT cells was significantly reduced in *Gsdmd*^*−/−*^ and *Caspase-11*^*−/−*^ mice following daily adenine feeding. These above-mentioned results confirm that Caspase-11/GSDMD contributes to proinflammatory and profibrogenic factors release and α-SMA expression in macrophages.

### Caspase-11/GSDMD in hematopoietic cells contribute to renal fibrosis in hyperuricemic nephropathy

**Fig. 6:**
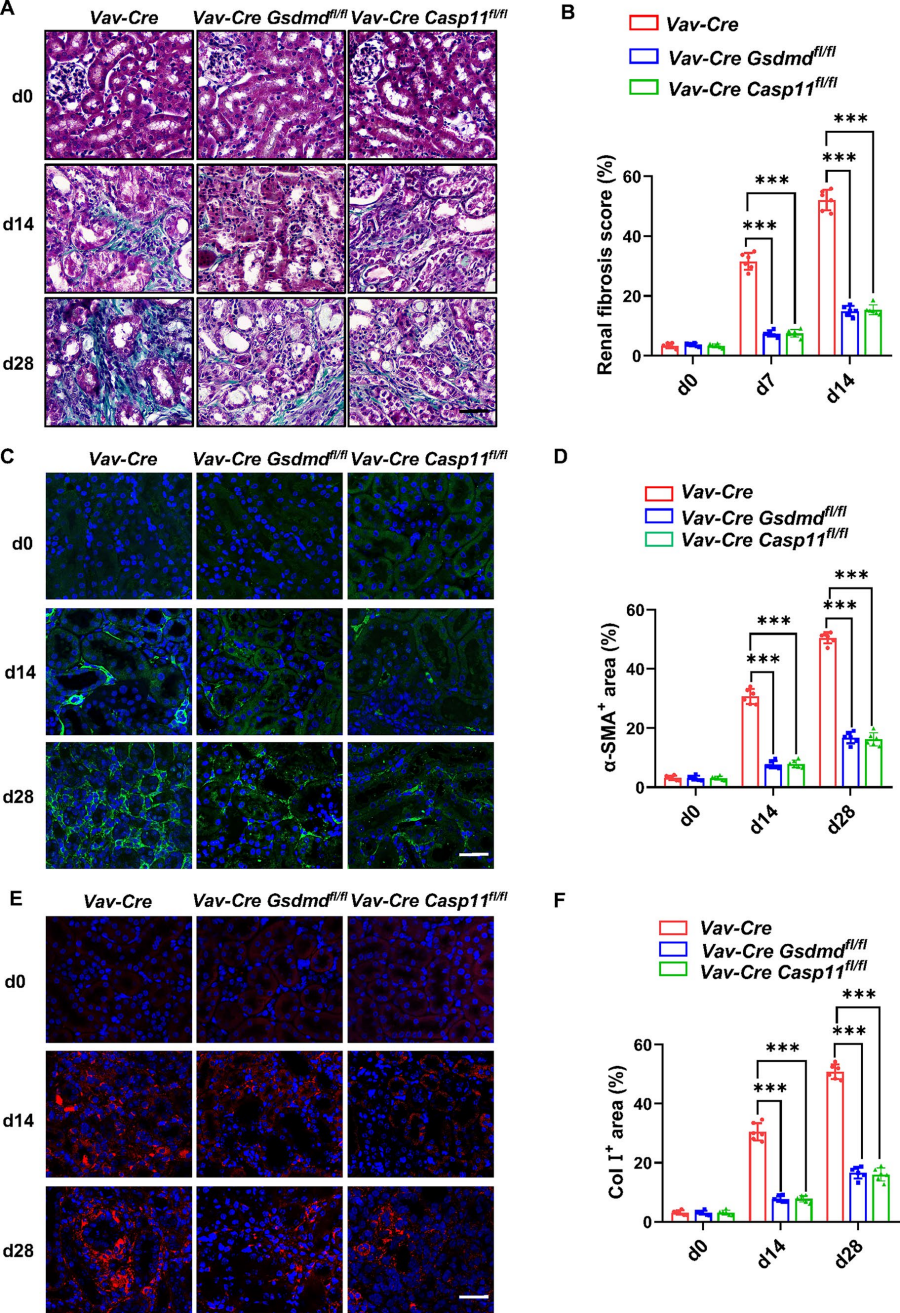
Specific deletion of *Caspase-11/Gsdmd* protected against the progression of hyperuricemic nephropathy by reduce fibrosis.** A** Representative images of Masson trichrome staining in mice kidney cortical tissue. Kidneys were harvested from *Vav-Cre* mice, *Vav-Cre Gsdmd*^*fl/fl*^* mice*, and *Vav-Cre Caspase-11*^*fl/fl*^ mice after daily adenine feeding for 0, 14, and 28 days. Scale bar = 100 μm. **B** Renal fibrosis scores assessed by Masson trichrome staining. *n* = 6. **C, E** Representative immunofluorescence images stained with α-SMA (**C**) and Col I (**E**) in mice kidney cortical tissue. Scale bar = 100 μm. **D, F** Quantitative analysis of α-SMA (**D**) and Col I (**F**) expression. *n* = 6. ***P < 0.001

In a preceding study, our team demonstrated that *Gsdmd* in bone marrow-derived neutrophils contributed to renal fibrosis in obstructive nephropathy [20]. To address whether GSDMD/Caspase-11 in hematopoietic cells takes responsibility for renal fibrosis in hyperuricemic nephropathy, we generated hematopoietic cells-specific *Gsdmd*-deficient mice and hematopoietic cells-specific *Caspase-11*-deficient mice by crossing *Gsdmd* floxed mice (*Gsdmd*^*fl/fl*^ mice) and *Caspase-11* floxed mice (*Caspase-11*^*fl/fl*^ mice) with Vav-Cre-expressing mice on a C57BL/6 background, respectively. Compared with WT mice (*Vav-Cre*), hematopoietic cells-specific *Gsdmd*-deficient mice (*Vav-Cre Gsdmd*^*fl/fl*^) and hematopoietic cells-specific *Caspase-11*-deficient mice (*Vav-Cre Caspase-11*^*fl/fl*^) showed a pronounced reduction in renal fibrosis after daily feeding of adenine for 14 days and 28 days (Fig. 6A, B). Consistently, the expression of α-SMA (Fig. 6C, D) and Col I (Fig. 6E, F) in the kidneys of *Vav-Cre Gsdmd*^*fl/fl*^ and *Vav-Cre Caspase-11*^*fl/fl*^ were significantly reduced after the same adenine dietary regimen. Altogether, these findings provide the compelling evidence that Caspase-11/GSDMD in hematopoietic cells is responsible for the progression of renal fibrosis in hyperuricemic nephropathy.

### Caspase-11/GSDMD played an essential role in NETs generation in hyperuricemic nephropathy

**Fig. 7:**
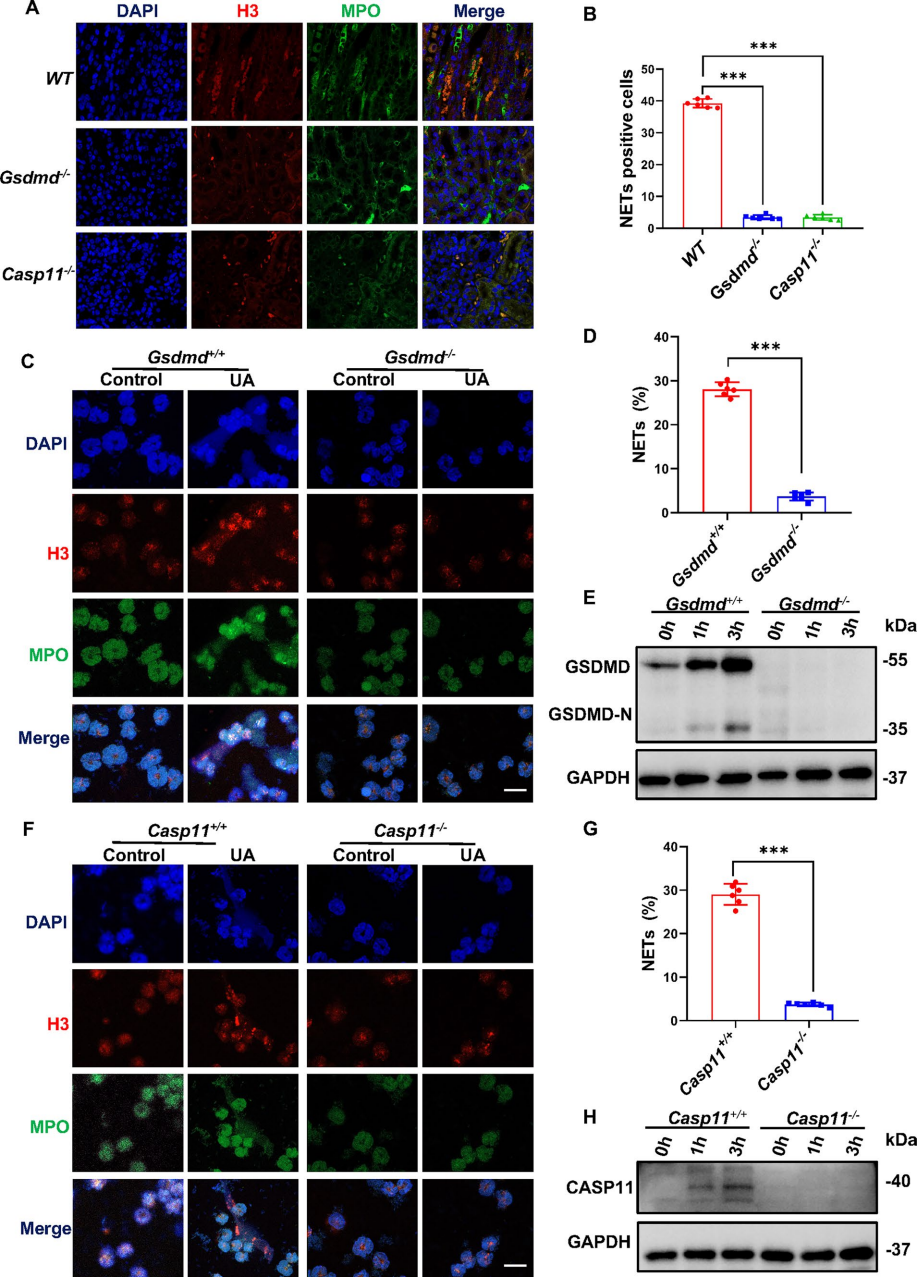
Deletion of *Caspase-11/Gsdmd* reduced NETs formation in vivo and in vitro. **A** Representative immunofluorescence images stained with Histone-H3 and MPO in mice kidney cortical tissue. Kidneys were harvested from WT mice, *Gsdmd*^*−/−*^ mice, and *Caspase-11*^*−/−*^ mice after daily adenine feeding for 14 days. Scale bar = 100 μm. **B** Quantification of neutrophils with Histone-H3 and MPO double-positive staining. *n* = 6. **C** Representative immunofluorescence images stained with Histone-H3 and MPO in neutrophils isolated from *Gsdmed*^+*/*+^ mice and *Gsdmd*^*−/−*^ mice and then were incubated with or without UA for 3 h. Scale bar = 50 μm. **D** Quantification of the percentage of Histone-H3 and MPO double-positive area. **E** Representative Western blot images showing GSDMD cleavage in neutrophils after incubation with UA for 1 h and 2 h. The knockdown efficiency of *Gsdmd* was confirmed. *n* = 6. **F** Representative images of immunofluorescence staining of Histone-H3 and MPO in neutrophils isolated from *Caspase-11*^+*/*+^ mice and *Caspase-11*^*−/−*^ mice and then were incubated with or without UA for 3 h. Scale bar = 50 μm. **G** Quantification of the percentage of Histone-H3 and MPO double-positive area. **H** Western blot showing Caspase-11 activation in neutrophils after incubation with UA for 1 and 2 h. The knockout efficiency of *Caspase-11* was confirmed. *n* = 6. ***P < 0.001

Our prior investigation established that Caspase-11/GSDMD actively participates in the generation of NETs, which promotes MMT and contributes to renal fibrosis [20]. To address the question of whether Caspase-11/GSDMD played a vital role in NETs generation in hyperuricemic nephropathy, we accessed the expression of myeloperoxidase (MPO) and histone H3 (H3) in the kidney tissue from *Gsdmd*^*−/−*^ mice, *Caspase-11*^*−/−*^ mice, and WT control mice. After daily adenine feeding for 14 days, neutrophils in the renal interstitium of WT mice were extensively labeled with MPO and H3, indicative of NETs generation (Fig. 7A, B). It is noteworthy that the release of NETs was abrogated in *Gsdmd*^*−/−*^ mice and *Caspase-11*^*−/−*^ mice.

To further validate whether GSDMD/Caspase-11 activation was involved in NETs generation in vitro, we isolated neutrophils from *Gsdmd*^*−/−*^, *Caspase-11*^*−/−*^, and WT mice. Bone marrow neutrophils were stimulated with uric acid for 0 h, 1 h, and 3 h. We observed that GSDMD/Caspase-11 were activated in a time-dependent manner after stimulation in *Gsdmd*^+*/*+^ and *Caspase-11*^+*/*+^ neutrophils (Fig. 7E, H). Immunofluorescence staining confirmed that uric acid treatment induced NETs formation in *Gsdmd*^+*/*+^ and *Caspase-11*^+*/*+^ neutrophils, which was prevented in both *Gsdmd*^*−/−*^ and *Caspase-11*^*−/−*^ neutrophils (Fig. 7C, D, F, G), corroborating our in vivo data. Collectively, these data provide robust support for the hypothesis that GSDMD/Caspase-11 contributes to the release of NETs from neutrophils in hyperuricemic nephropathy.

### NETs promoted α-SMA production in macrophages

**Fig. 8:**
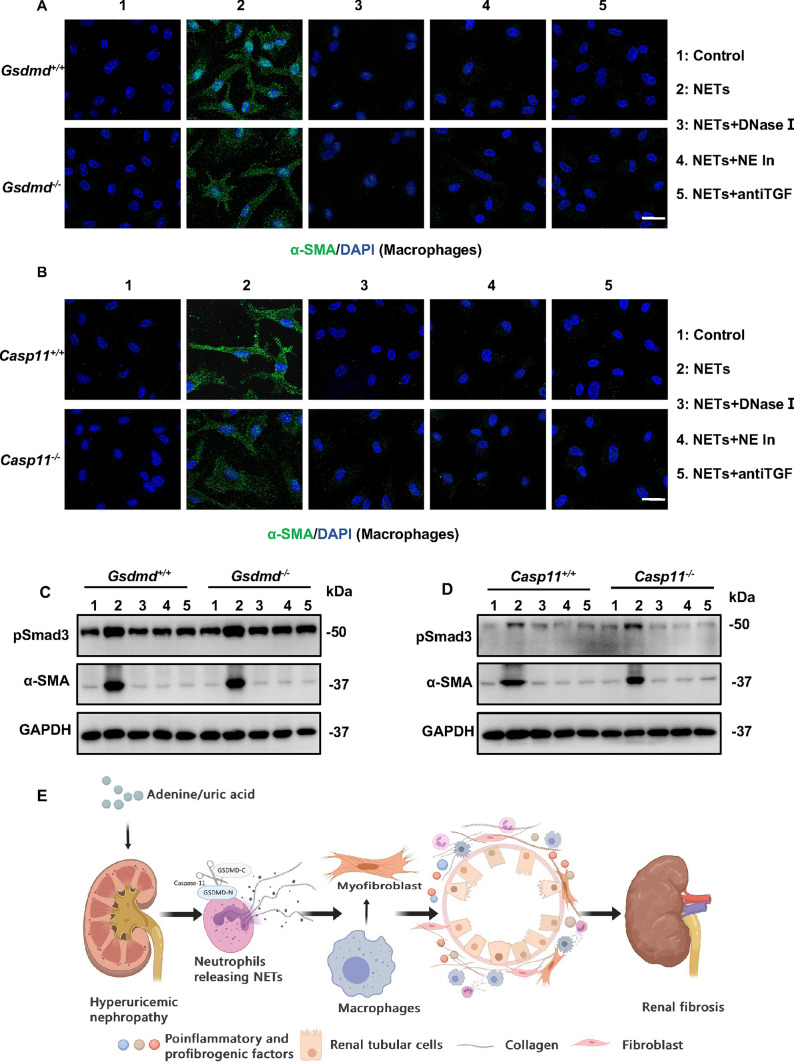
NETs promoted α-SMA production in macrophages in vitro. **A, B** Representative immunofluorescence images showing the expression of α-SMA in microphages. Microphages were isolated form *Gsdmed*^+*/*+^ mice, *Gsdmd*^*−/−*^mice, *Caspse-11*^+*/*+^ mice, and *Caspase-11*^*−/−*^mice, and were then treated with NETs, NETs plus DNase I, NETs plus NE inhibitor or NETs plus anti-TGF-β1 antibody for 96 h. Scale bar = 50 μm. **C, D** Representative Western blot results showing the expression levels of α-SMA and its regulator, pSmad3, in macrophages. *n* = 4. **E** Schematic model: GSDMD/Caspase-11 contributes to the progression of hyperuricemic nephropathy by promoting NETs formation

To investigate the interplay between NETs and macrophages in vitro, we induced NETs formation using uric acid in neutrophils isolated from WT mice. Purified NETs was collected and utilized to stimulate isolated macrophages, aiming to elucidate whether NETs could influence the production of α-SMA. As expected, NETs derived from WT neutrophils significantly augmented the expression of α-SMA in macrophages isolated from *Gsdmd*^*−/−*^, *Caspase-11*^*−/−*^, and WT mice (Fig. 8A, B). Consistently, disruption of NETs generation by DNase I or neutrophil elastase inhibitor was able to reduce α-SMA production. In addition, NETs-triggered α-SMA expression in macrophages was reduced by anti-TGF-β1 antibody treatment. These findings were further determined by Western blot analysis (Fig. 8C, D). Taken together, these results demonstrate that NETs play a pivotal role in promoting α-SMA production in macrophages.

## Discussion

Globally, the prevalence of CKD is estimated at 13.4%, and this figure is on the rise [28]. Although there is mounting evidence suggesting that hyperuricemia independently poses a risk factor for renal fibrosis and progressive CKD, the underlying mechanisms remain incompletely understood [2]. In our current study, we identified Caspase-11/GSDMD as a critical role in progressive hyperuricemic nephropathy, as its deletion significantly reduced the infiltration of neutrophils and macrophages, NETs formation, α-SMA expression in macrophages, and renal fibrosis (Fig. 8E). Specific deletion of *Caspase-11/Gsdmd* in hematopoietic cells remarkably prevented renal fibrosis in hyperuricemic nephropathy. We further revealed that Caspase-11/GSDMD-dependent NETs is critically involved in the pathogenesis of hyperuricemic nephropathy.

GSDMD, a well-known pore-forming protein that is crucial for the execution of pyroptosis, was recently identified as a central regulator of NET formation [18, 19]. Subsequently, evidence of the vital role of GSDMD-dependent NETs in diseases emerged. Our prior study demonstrated that GSDMD was required for the generation of NETs in obstructive nephropathy [20]. Miao et al*.* indicated that GSDMD actively contributed to NETs formation and mtDNA release, promoting systemic lupus erythematosus pathogenesis [29]. In sickle cell disease, inhibition of caspase11 or GSDMD abrogated vaso-occlusion by blocking NETs formation [17]. In addition, excessive release or impaired degradation of NETs has been proposed to contribute to various kidney diseases, including AKI and autoimmune kidney diseases [16]. Patients with lupus nephritis tend to have defects in degrading NETs, which prolongs the exposure time of self-antigens to host cells, thereby contributing to organ damage [30, 31]. In antineutrophil cytoplasmic autoantibody (ANCA)-associated vasculitis, NETs, which could be detected in patients’ blood and kidney biopsy samples, was assumed to contribute to ANCA production, exacerbating renal injury [32, 33]. A recent article indicated that the elimination of NETs significantly attenuates glomerular endothelial cell injury and alleviates the development of diabetic kidney disease [34]. However, research on Caspase-11/GSDMD and NETs in the context of hyperuricemic nephropathy is lacking. In this study, we observed that Caspase-11/GSDMD activation was associated with renal fibrosis, inflammatory cell infiltration, proinflammatory and profibrogenic factors expression, and α-SMA production. Moreover, we uncovered that Caspase-11/GSDMD axis played critical role in the progression of hyperuricemic nephropathy through mediating the generation of NETs in vivo and in vitro.

It is widely believed to be a wound-healing process in response to renal injury, in which numerous immune cells infiltrate into both the glomerulus and interstitium. Unequivocal evidence indicates that neutrophils are recruited within the very first 24 h following renal injury, followed by monocyte recruitment [35]. Indeed, our current study found that the numbers of neutrophils peaked at day 14 in mice with an adenine diet, while macrophages peaked subsequently. The intricate partnership between neutrophils and macrophages has been an area of interest and challenge. Recently, NETs were demonstrated to be involved in the coordinated interplay between neutrophils and macrophages. On the one hand, NETs could trigger macrophage polarization toward a reparative phenotype. Josefs et al*.* suggested that NETs formation induced a proinflammatory macrophage phenotype, which in turn propagates inflammation in the plaques of diabetic mice [36]. A more recent study from Liu et al*.* revealed that NETs impeded macrophage differentiation into the anti-inflammatory phenotype by inactivating the TGF-β1 signaling pathway, further aggravating tissue dysfunction [37]. Consistent with previous research, we also observed that hyperuricemia resulted in increased production of proinflammatory and profibrogenic factors, such as TGF-β1 and IL-1β [6, 8]. The critical role of the TGF-β1 signaling pathway in tubulointerstitial fibrosis is well established [29, 38]. On the other hand, NETs could also prime macrophages for cytokine release. In the context of atherosclerosis, neutrophils have the capacity to prime macrophages for the transcription of IL-1β by releasing NETs [39]. NETs plays a contributory role in tissue remodeling and fibrosis in the lung by releasing externalized histones, thereby inhibiting the production of anti-fibrotic IL-27 by macrophages [40]. In addition, Chen et al*.* identified a novel mechanism by which NETs induce pyroptosis in macrophages by releasing HMGB1, thereby amplifying the inflammation following infection [41]. Given the impact of neutrophil–macrophage cooperation on inflammation and fibrosis, we found that NETs increased the expression levels of α-SMA in macrophages, in line with the previous investigations, indicating that NETs boosted fibronectin expression in macrophages [42].

More interestingly, we discovered that Caspase-11/GSDMD in hematopoietic cells determined the progress of hyperuricemic nephropathy. A previous study from our group identified that specific deletion of *Gsdmd* in neutrophils rather than macrophages provide renoprotection after unilateral ureteral obstruction [20]. During sepsis, GSDMD activation in neutrophils, but not monocytes, is relevant to multiple organ dysfunction [43]. In this study, we found uric acid treatment induced NETs formation in neutrophils isolated from the bone marrow of WT mice, which was inhibited by deletion of *Gsdmd* or *Caspase-11*. These results supported our hypothesis that Caspase-11/GSDMD in neutrophils are relevant to the progression of hyperuricemic nephropathy. However, further study is needed to elucidate the exact type of immune cell that takes the responsibility.

In summary, our study demonstrated that specific deletion of *Caspase-11/Gsdmd* in hematopoietic cells attenuated the development of hyperuricemic nephropathy. This effect was related to the blockade of NETs formation in neutrophils and the reduction of α-SMA expression in macrophages. Thus, targeting Caspase11/GSDMD-dependent NETs may hold therapeutic potential for treating progressive hyperuricemic nephropathy.

### Supplementary Information

Below is the link to the electronic supplementary material.

Supplementary file1 (PDF 1689 KB)

## Data Availability

All data needed to evaluate the conclusions in the paper are presented in the paper or the Supplementary materials. Any additional information required to reanalyze the data reported in this paper is available from the lead contact upon reasonable request.

## Abbreviations

CKD: Chronic kidney disease
Col I: Collagen I
NETs: Neutrophil extracellular traps
GSDMD: Gasdermin D
α-SMA: α-Smooth muscle actin
IL-1β: Interleukin-1beta
TNFα: Tumor necrosis factor-alpha
TGF-β1: Transforming growth factor-beta1
HMGB1: High-mobility group box-1
WT: Wild type
MMT: Macrophage-to-myofibroblast transition
H3: Histone
MPO: Myeloperoxidase
ANCA: Antineutrophil cytoplasmic autoantibody
