# Supplementary material for: Caspase-11/GSDMD contributes to the progression of hyperuricemic nephropathy by promoting NETs formation
Source: Cell Mol Life Sci. 2024 Mar 4;81(1):114. doi: 10.1007/s00018-024-05136-z (PMC10912150; doi:10.1007/s00018-024-05136-z)
Supplement: Supplementary file 1 — Supplementary file1 (PDF 1689 KB) [file 18_2024_5136_MOESM1_ESM.pdf]

Supplementary materials

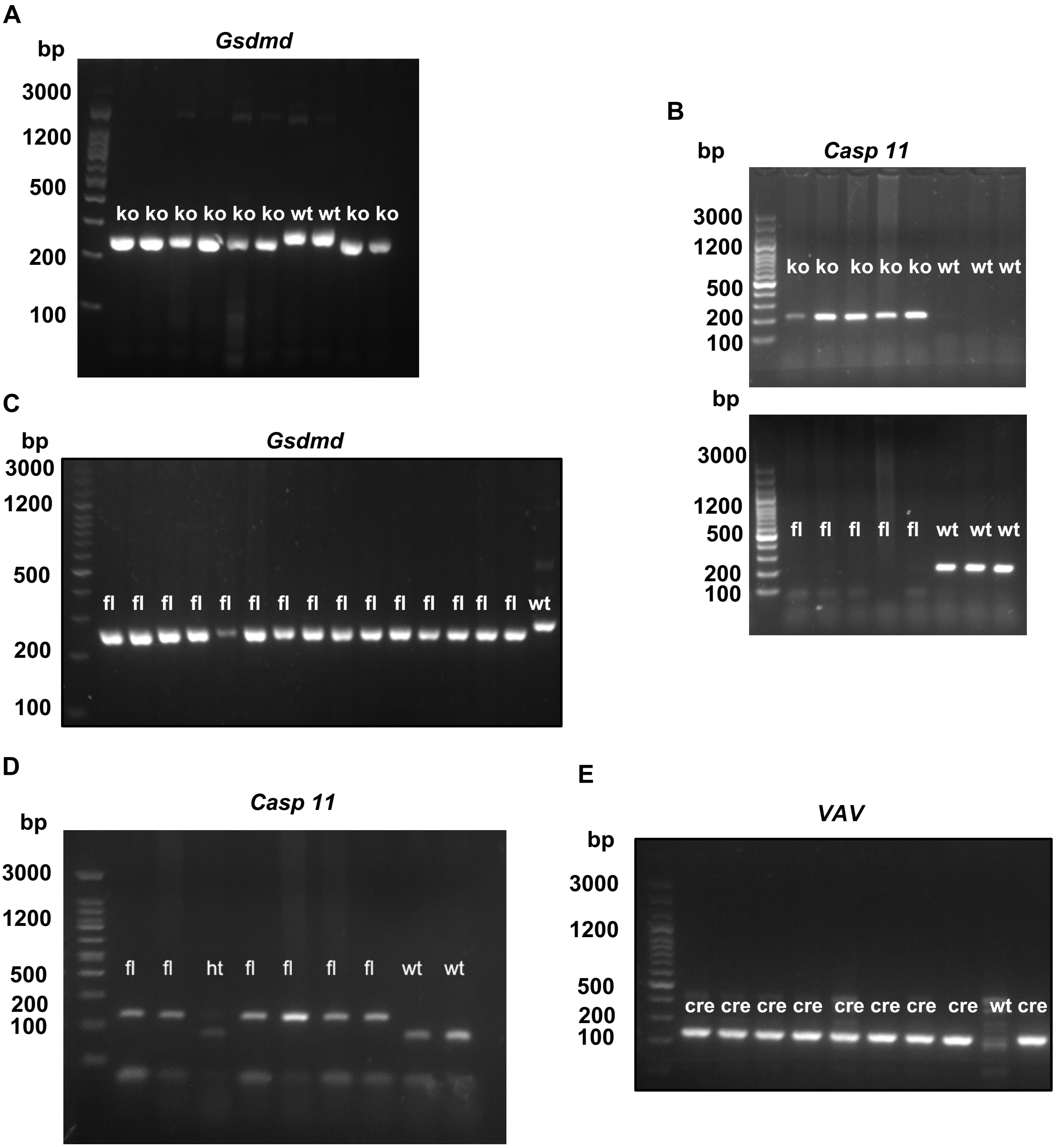

Figure S1 Identification of gene-editing mice. (A) Representative DNA gel of ***Gsdmd*$^{-/-}$ mice and *Gsdmd*$^{+/+}$ mice. (B) Representative DNA gel of *Caspase-11*$^{-/-}$ mice and *Caspase-11*$^{+/+}$ mice. (C) Representative DNA gel of *Gsdmd*$^{fl/fl}$ mice and *Gsdmd*$^{wt/wt}$ mice. (D) Representative DNA gel of *Caspase-11*$^{fl/fl}$ mice, *Caspase-11*$^{wt/wt}$ mice, and heterozygotes. (E) Representative DNA gel of *Vav-Cre* mice and wildtype pups.**

Supplementary materials

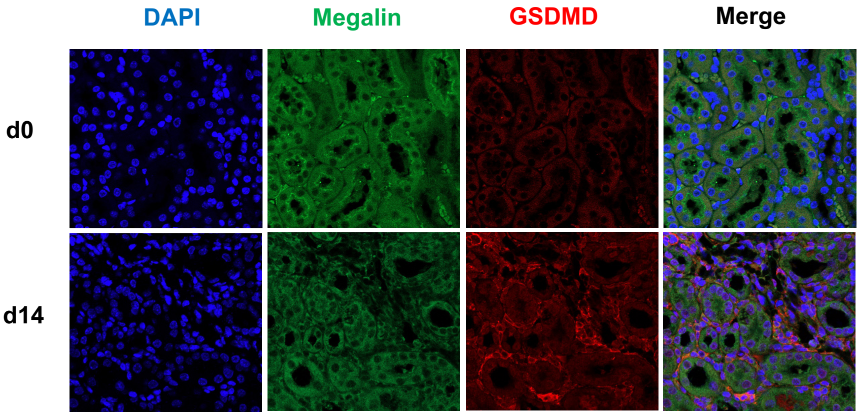

Figure S2 Representative images of immunofluorescence staining of kidney sections from wild-type mice on day 14 after daily feeding of adenine, showing the expression of megalin (green) and GSDMD (red). DAPI (blue) was used for nuclear staining.
